# Supplementary material for: Exchange biased anomalous Hall effect driven by frustration in a magnetic kagome lattice
Source: Nat Commun. 2020 Jan 28;11:560. doi: 10.1038/s41467-020-14326-9 (PMC6987238; doi:10.1038/s41467-020-14326-9)
Supplement: Supplementary file 1 — Supplementary Information [file 41467_2020_14326_MOESM1_ESM.pdf]

# Supplementary Information
# Exchange biased anomalous Hall effect driven by frustration in a magnetic kagome lattice

Ella Lachman*[1,2], Ryan A. Murphy[3], Nikola Maksimovic[1,2], Robert Kealhofer[1,2], Shannon Haley[1,2], Ross D. McDonald[4], Jeffrey R. Long[2,3,5] and James G. Analytis[1,2]*

[1]*Department of Physics, University of California, Berkeley, California 94720, USA*

[2]*Materials Science Division, Lawrence Berkeley National Laboratory, Berkeley, California 94720, USA*

[3]*Department of Chemistry, University of California, Berkeley, California 94720, USA*

[4]*Los Alamos National Laboratory, Los Alamos, New Mexico 87545, USA*

[5]*Department of Chemical and Biomolecular Engineering, University of California, Berkeley, California 94720, USA*

* corresponding author: ellal@berkeley.edu

## Supplementary Note 1. COMPLEX ENERGY LANDSCAPE EXPLAINING MAGNETIZATION HYSTERESIS

The magnetic hysteresis loops presented in the main text (Fig. 4(a)) show a non-conventional shape of the hysteresis. We believe this can be explained by realizing that the energy landscape of the system is more complex than a simple ferromagnet. As the sample is cooled down below $T_c = 175$ K, the system is not strictly ferromagnetic with out-of-plane magnetization, but already glassy (see Fig. 6(d,e)) possibly with in-plane canting of the spins. This magnetization has a complex landscape with a global minimum at zero magnetization at zero field (Fig. 1(g)). When tilted with applied magnetic field (Fig. 1(d,j)), the local minimum at $\pm M_0$ becomes a global one, and the system becomes magnetized. As the temperature is lowered towards $T_G = 125$ K the glass becomes stiffer, manifested in the $\pm M_0$ minima becoming deeper, creating a meta-stable state at low fields (Fig. 1 (h)). For temperatures well below $T_G$, the $M = 0$ minimum is shallow compared to the $\pm M_0$ minima, effectively reproducing the ferromagnetic energy landscape, yielding the classic ferromagnetic hysteresis loop.

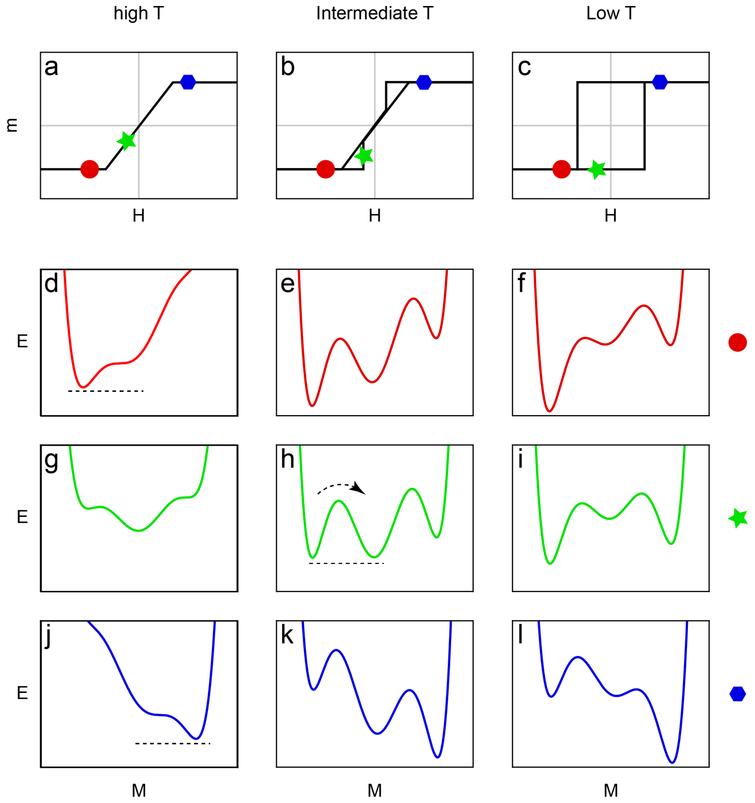


Supplementary Figure 1. Complex magnetic energy landscape explaining the hysteresis loops shown in Fig. 4(a). (a-c) The schematic shape of the magnetic hysteresis loops presented in Fig. 4(a) for the temperature range of $T_c > T > T_G$ (a), $T \leq T_G$ (b) and $T < T_G$ (c). On each of these there are three marks indicating the amplitude of the magnetic field: red circle for negative high fields, green star for negative low fields and a blue hexagon for positive high fields. For each of these fields, the three schematic energy landscapes are shown for each of the temperature ranges. Low temperatures (f,i,l), intermediate temperatures (e,h,k) and high temperatures (d,g,j). In (h) an arrow marks the meta-stable state manifesting as the jump seen in (b) where the green star is.

## Supplementary Note 2. NON-TRIVIAL DYNAMICS AT LOW TEMPERATURES

A spin glass phase is usually characterized by magnetization relaxation, and by a frequency dependence in AC susceptibility. However, in our case the spin glass coexists with a strong ferromagnetic order with a higher transition temperature. As a complementary low-temperature demonstration of the non-trivial dynamics of $Co_3Sn_2S_2$, we show here a field-sweep-rate dependence of the hysteresis loop for a zero-field cooled sample.

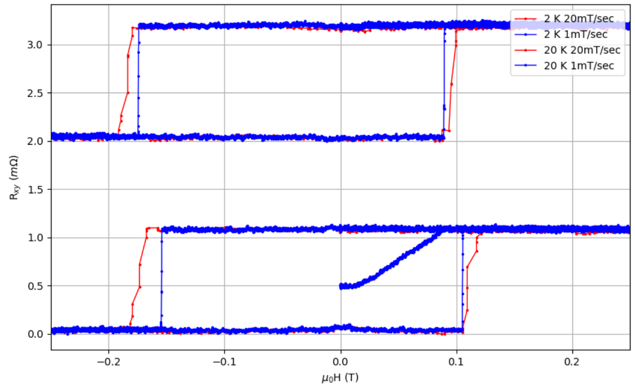


Supplementary Figure 2. Hall resistance as a function of applied magnetic field at temperatures of 2 K (lower) and 20 K (higher). The two field sweeping rate are 20 mT (200 Oe) per second in red, and 1 mT (10 Oe) per second in blue. The curves are shifted for clarity and for easy comparison. As is expected for a slow-dynamics phase such as spin glass, sweeping the field faster results in a loop with a larger area, as the phase responds slowly to the varying field and remains in the down (up) polarized state when sweeping the field up (down).

## Supplementary Note 3. ADDITIONAL MAGNETIZATION DATA

Field cooled (50 mT) c-axis magnetization measurements for our samples are in agreement with previous measurements showing $\sim 0.33\mu_B$ per Co atom.

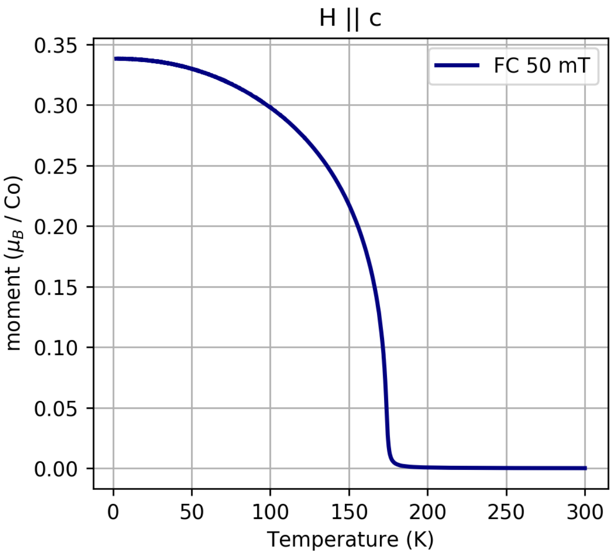


Supplementary Figure 3. Out of plane (c-axis) field cooled (50 mT) magnetization vs. temperature measurement.

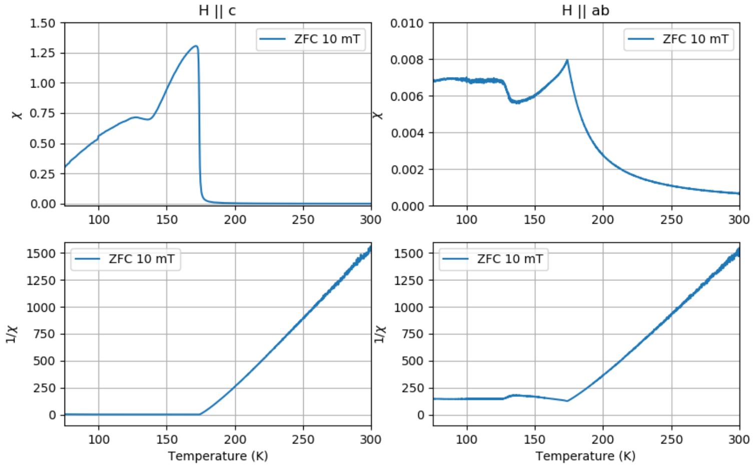


Supplementary Figure 4. $\chi$ and $1/\chi$ for a sample cooled in zero field and measured warming up from 2 K in a magnetic field $\mu_0 H = 10$ mT.

## Supplementary Note 4. LOW FIELD HEAT CAPACITY MEASUREMENT

Heat capacity measurements similar to those presented in Fig. 5(c) performed at a lower field ($\mu_0 H = 10$ mT). As in the original measurement and unlike in the case of the magnetization, the low field does not reveal a $T_G = 125$ K transition.

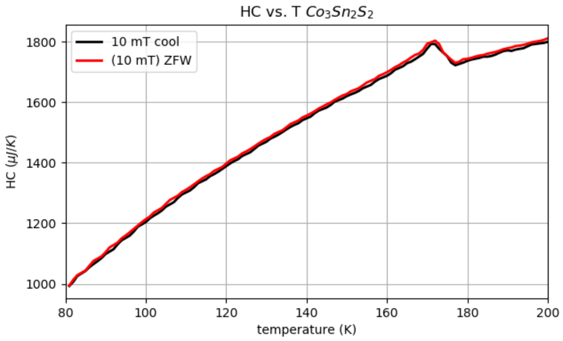


Supplementary Figure 5. Single crystal heat capacity as a function of temperature for $80 - 200$ K. These measurements reveal no feature at 125 K.

## Supplementary Note 5. PULSED HIGH MAGNETIC FIELD QUANTUM OSCILLATIONS

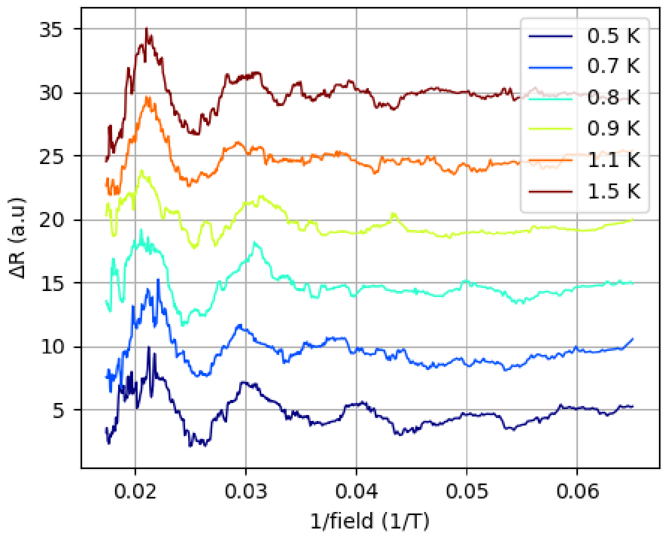


Supplementary Figure 6. SdH oscillations amplitudes obtained by subtracting a cubic polynomial from the magneto-resistance data acquired at the pulsed high magnetic field lab.

## Supplementary Note 6. AS-TAKEN TRANSPORT MEASUREMENT

Due to the nature of the manipulation performed on the transport data to account for mixing, i.e removing a constant ratio of $R_{xx}$ from $R_{xy}$ data, we show here the "as measured" data of both $R_{xy}$ and $R_{xx}$ of Figures 2,3 and 4. These are shown so that readers would be able to judge possible artifacts introduced by this approach.

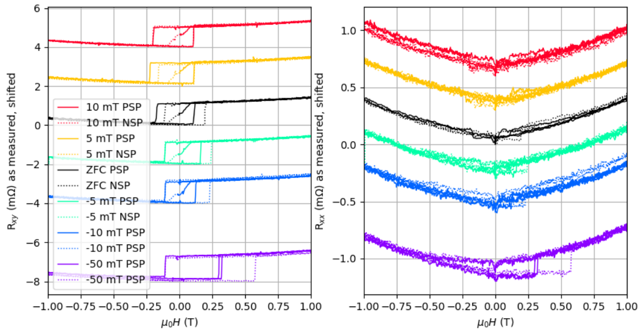


Supplementary Figure 7. Original data from spontaneous exchange bias measurement shown in Fig. 2. The data is shifted vertically for clarity.

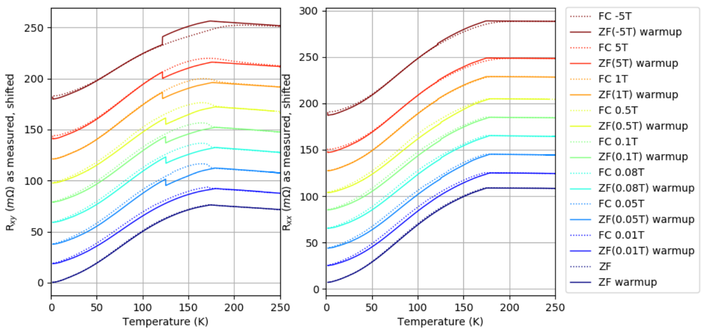


Supplementary Figure 8. Original data from zero-field warming up measurement shown in Fig. 3. The data is shifted vertically for clarity.

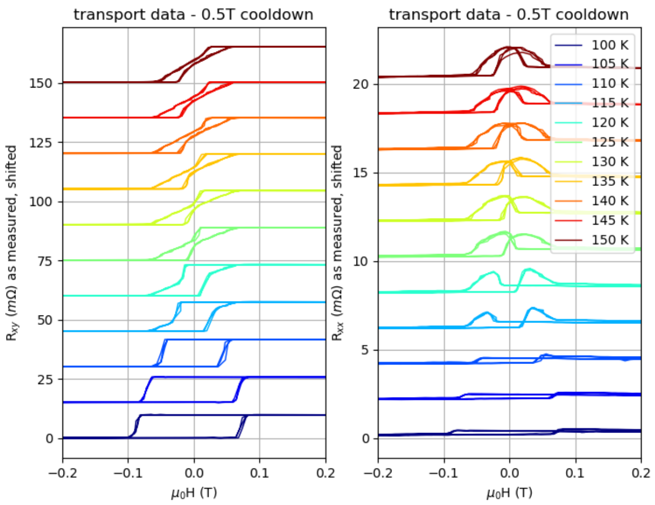


Supplementary Figure 9. Original data from magnetic field sweep measurement shown in Fig. 4(b). The data is shifted vertically for clarity.

## Supplementary Note 7. OTHER SAMPLES

As stated in the main text, our results span different samples from different, independent crystal growths. Figure 10 shows the FC-ZFW protocol on a different sample, showing the $R_{xy}$ anomaly. Figure 11 shows the Exchange Bias present in the sample at 2 K after field cooling in $\mu_0 H_{cool} = 2$ T.

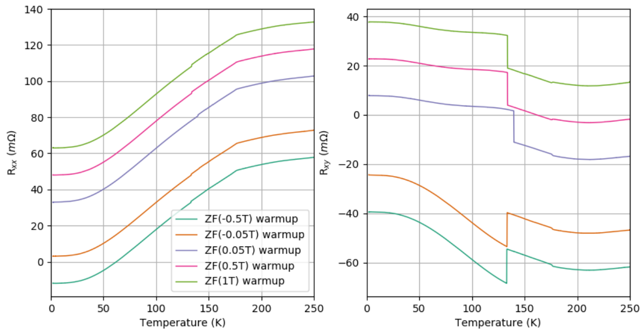


Supplementary Figure 10. Resistance as function of temperature for zero field warming up after in-field cooling of a different sample. On the zero-field warming up curves, the anomaly is evident as a jump to a different value. For cooling in positive (negative) field, the jump is to a lower (higher) value.

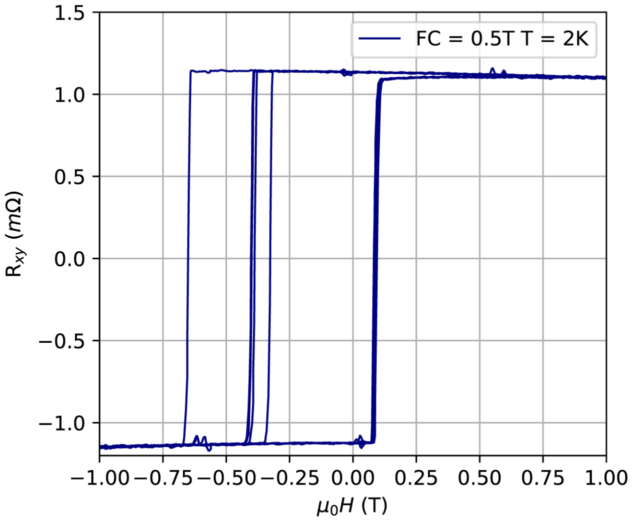


Supplementary Figure 11. Resistance as function of field for a sample cooled in-field. This is the same protocol as described in the inset of Fig.1(b) in the main text.
